# 1324. Impact of Implementation of CSF PCR Panel in Patients with Meningitis

**DOI:** 10.1093/ofid/ofad500.1162

**Published:** 2023-11-27

**Authors:** Eric Hsu, Nirusha Abeydeera, Samia Faiz, Napatkamon Ayutyanont, Robyn Meadows, Sudhakar Mallela

**Affiliations:** Riverside Community Hospital, cerritos, California; Riverside Community Hospital/ HCA Healthcare, Riverside, California; Riverside Community Hospital, cerritos, California; HCA Healthcare, Riverside, California; Riverside Community Hospital, cerritos, California; HCA Healthcare, Nashville, TN;Riverside Community Hospital, Riverside, CA; University of California, Riverside, CA, Riverside, California

## Abstract

**Background:**

Cerebrospinal fluid (CSF) Polymerase chain reaction (PCR) panel is routinely used for etiologic diagnosis of meningitis. While its role in this setting has been proven, there is no data on its benefits on duration of antibiotic use and hospital length of stay.

**Methods:**

Retrospective cohort study was performed to compare meningitis patients who received the CSF PCR panel versus those who did not in a large database of HCA hospitals across the United States.

Data was collected from January 2014 to May 2022 on all patients diagnosed with meningitis with a CSF WBC greater than 10. Using logistic regression and negative binomial regression, the study compared utility of CSF PCR panel use versus non-use with regards to length of stay, antibiotic duration, resolution of fever and leukocytosis, and hospital readmission rate.

Demographics by Panel Use

**Figure:**
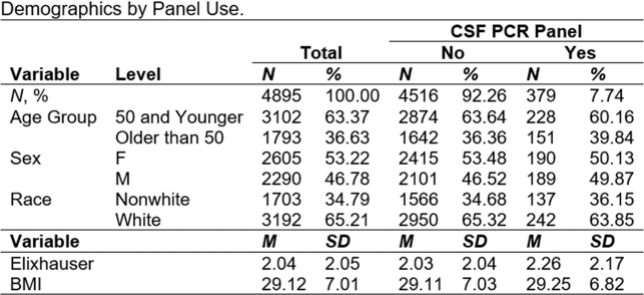

A total of 4895 patients were included in the study, of which 379 received the CSF PCR Meningitis Panel.

**Results:**

A total of 4895 distinct patient encounters were included, of whom 379 patients had the CSF PCR panel. CSF PCR use was associated with a significant decrease in readmission rates within 30 days (x2 5.64, p< 0.05) and had decreased log count days of antibiotic duration (x2 17.56, p< 0.0001) with a mean duration of antibiotic use of 73.91 hours in CSF PCR panel patients versus 82.05 hours. Panel use was not significantly associated with length of stay (x2 0.96, p 0.328). The likelihood of fever resolution and leukocytosis resolution was the same for those who received the CSF PCR Panel versus those who did not.

Antimicrobial Medications Received by Panel Use

**Figure:**
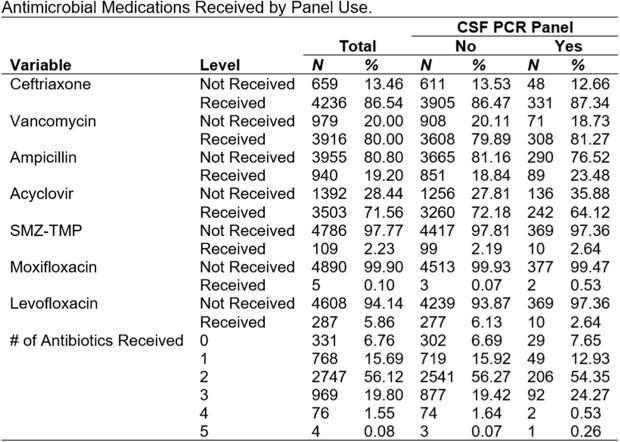

Antimicrobial Duration and Clinical Presentation by Panel Use

**Figure:**
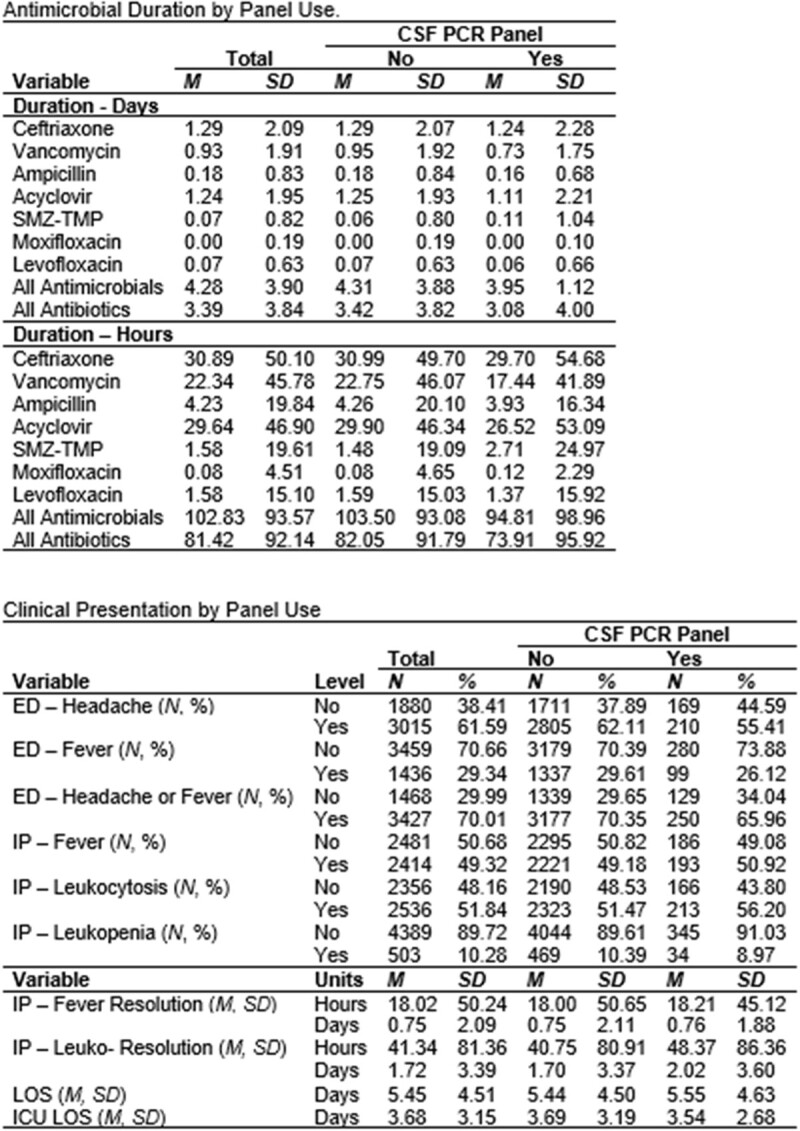

There was a decrease in antibiotic use of 73.91 hours in CSF PCR panel patients versus 82.05 hours. There was also a significant decrease in hospital readmission rates within 30 days

**Conclusion:**

CSF PCR panel benefits included reduced antibiotic duration of use and reduced 30-day readmission rates. Fever resolution and leukocytosis or leukopenia resolution was not impacted by CSF PCR panel use.

**Disclosures:**

**All Authors**: No reported disclosures

